# Automatic segmentation of white matter hyperintensities and correlation analysis for cerebral small vessel disease

**DOI:** 10.3389/fneur.2023.1242685

**Published:** 2023-07-27

**Authors:** Bin Xu, Xiaofeng Zhang, Congyu Tian, Wei Yan, Yuanqing Wang, Doudou Zhang, Xiangyun Liao, Xiaodong Cai

**Affiliations:** ^1^Department of Neurosurgery, Shenzhen Second People's Hospital, The First Affiliated Hospital of Shenzhen University, Shenzhen, Guangdong, China; ^2^Shenzhen University School of Medicine, Shenzhen, Guangdong, China; ^3^Shenzhen Institute of Advanced Technology, Chinese Academy of Sciences, Shenzhen, China; ^4^Brain Cognition and Brain Disease Institute, Shenzhen Institutes of Advanced Technology, Chinese Academy of Sciences, Shenzhen, China

**Keywords:** cerebral small vessel disease, white matter hyperintensity, deep encoder-decoder structure, medical 3D segmentation, correlation analysis

## Abstract

**Objective:**

Cerebral white matter hyperintensity can lead to cerebral small vessel disease, MRI images in the brain are used to assess the degree of pathological changes in white matter regions. In this paper, we propose a framework for automatic 3D segmentation of brain white matter hyperintensity based on MRI images to address the problems of low accuracy and segmentation inhomogeneity in 3D segmentation. We explored correlation analyses of cognitive assessment parameters and multiple comparison analyses to investigate differences in brain white matter hyperintensity volume among three cognitive states, Dementia, MCI and NCI. The study explored the correlation between cognitive assessment coefficients and brain white matter hyperintensity volume.

**Methods:**

This paper proposes an automatic 3D segmentation framework for white matter hyperintensity using a deep multi-mapping encoder-decoder structure. The method introduces a 3D residual mapping structure for the encoder and decoder. Multi-layer Cross-connected Residual Mapping Module (MCRCM) is proposed in the encoding stage to enhance the expressiveness of model and perception of detailed features. Spatial Attention Weighted Enhanced Supervision Module (SAWESM) is proposed in the decoding stage to adjust the supervision strategy through a spatial attention weighting mechanism. This helps guide the decoder to perform feature reconstruction and detail recovery more effectively.

**Result:**

Experimental data was obtained from a privately owned independent brain white matter dataset. The results of the automatic 3D segmentation framework showed a higher segmentation accuracy compared to nnunet and nnunet-resnet, with a *p*-value of <0.001 for the two cognitive assessment parameters MMSE and MoCA. This indicates that larger brain white matter are associated with lower scores of MMSE and MoCA, which in turn indicates poorer cognitive function. The order of volume size of white matter hyperintensity in the three groups of cognitive states is dementia, MCI and NCI, respectively.

**Conclusion:**

The paper proposes an automatic 3D segmentation framework for brain white matter that achieves high-precision segmentation. The experimental results show that larger volumes of segmented regions have a negative correlation with lower scoring coefficients of MMSE and MoCA. This correlation analysis provides promising treatment prospects for the treatment of cerebral small vessel diseases in the brain through 3D segmentation analysis of brain white matter. The differences in the volume of white matter hyperintensity regions in subjects with three different cognitive states can help to better understand the mechanism of cognitive decline in clinical research.

## 1. Introduction

Cerebral small vessel disease of the brain refers to blood vessels affecting the arteries or veins of the cerebellum. This type of disease causes dysfunction of the tiny circulation from the small vessels to the small vessels, which can cause neurological dysfunction and impaire brain function and cognitive impairment. The white matter is made up of nerve fibers and neuronal axons responsible for transmitting information between different brain regions (1). The white matter requires a steady supply of blood through small blood vessels to maintain normal physiological functions (2). Cerebrovascular disease can cause a reduction or interruption of blood flow to these vessels, leading to the development of white matter hyperintensities that can be diagnosed through MRI image (3, 4).

The current clinical tools for detecting white matter abnormalities include observing MRI images (5), using neural networks to automatically segment and classify MRI images to determine the presence of abnormalities, analyzing the chemical composition of brain regions using magnetic resonance spectroscopy (6, 7). The use of 3D medical images can enhance clinical visualization during patient treatment (8), deep learning segmentation algorithms have demonstrated superior performance in segmenting large collections of data. In recent years, 3D image segmentation algorithms based on deep learning have shown superior performance in clinical medicine in recent years (9, 10).

In order to help physicians diagnose small vessel diseases more accurately in clinical work, automatic 3D segmentation of cerebral white matter hyperintensities provides a scientific basis for clinical treatment of small vessel diseases by analyzing white matter morphology. In order to enhance the detection of cerebral white matter hyperintensities, this paper proposes an automatic 3D segmentation framework, which avoids incomplete segmentation and over-segmentation in the process of segmenting cerebral white matter. In order to more comprehensively understand the difference of cerebral white matter hyperintensities volume in three cognitive states, this paper explores the correlation between cerebral white matter hyperintensities volume and cognitive assessment coefficient.

For brain white matter hyperintensities segmentation, we propose a deep multi-mapping encoder-decoder structure as an automatic 3D segmentation framework for brain white matter. A 3D residual mapping function is introduced in the overall segmentation framework. The residual function is widely used in image segmentation algorithms, which is known for its ability to solve the address information loss and ambiguity problem in image segmentation algorithms. The encoder-decoder structure is composed of multiple convolutional and pooling layers, which can result in missing image information due to layer-by-layer compression and downsampling, it leads to efficiency degradation and gradient explosion, due to the network's increased depth. To avoid this situation, this paper proposes an automatic 3D segmentation framework adapted to the white matter hyperintensities.

In this paper, we propose a three-dimensional Multi-layer Cross-connected Residual Mapping Module (MCRCM) in the encoding stage. The MCRCM Module can enhance the feature depth and width in the encoder stage, by adding multiple cross-connected residual structures, features at different levels can be cross-connected and fused, it enhances the feature representation capability and feature discriminability of the brain white matter hyperintensities. The 3D Spatial Attention Weighted Enhanced Supervision Module (SAWESM) is introduced in the decoding stage to upsample and reconstruct the features extracted by the 3D encoder, adding 3D spatial attention enhanced structures, which can adaptively adjust the feature weights and responses according to the spatial distribution of the features. The addition of a 3D spatial attention enhancement structure allows for adaptive adjustment of feature weights and responses according to the spatial distribution of the features, it enhances the supervisory hyperintensities through the 3D spatial attention mechanism, facilitating the convergence and generalization of the network.

The contributions of this paper are as follows:

1. The 3D automatic segmentation framework proposed in this paper achieves accurate segmentation of white matter hyperintensities, which helps to identify brain changes associated with cerebrovascular diseases and cognitive impairment.
2. The proposed MCRCM model enhances the medical feature representation capability of the model by extracting feature semantic information at different levels, while the proposed SAWESM model improves the medical segmentation accuracy of the model by capturing image boundary information and detail information during reconstruction.
3. Through correlation analysis and multiple comparisons of cognitive function coefficients, we found that the cognitive function coefficients in the three cognitive states showed strong significance, MMSE and MoCA showed negative correlation with brain white matter hyperintensities.

## 2. Related work

An adaptive fully dense (AFD) neural network is proposed for CT image segmentation (11). A hybrid 3D residual network with squeeze and excitation modules is proposed for volume segmentation in computerized tomography (CT) scans (12). 3D visual explanations using extended *post-hoc* interpretability techniques is analyzed for 3D brain tumor segmentation models (13). Wu proposed a new frame interpolation-based slice interpolation method to improve the segmentation accuracy of anisotropic 3D medical images (14). An improved template of fuzzy c-means (FCM) is proposed for 3D medical volume segmentation and suggested parallel implementation using image processing units (15).

A method based on 2D registration is proposed to gradually propagate labels between consecutive 2D slices and used 3D UNet to leverage volume information, to alleviate the burden of manual annotation (16). A novel multi-path densely connected convolutional neural network is proposed to automatically segment gliomas of unknown size, shape, and position (17). An automatic segmentation method based on deep learning is proposed to solve the problem of developing target localization pipelines in DBS surgery (18). A comprehensive system is proposed for detecting, measuring, analyzing the location of aneurysms on three-dimensional DS images (19). 3D contextual residual network is proposed for precise segmentation of 3D medical images, which consists of an encoder, segmental decoder, and contextual residual decoder (20).

Dilated Transformer is proposed to enlarge the receptive field without involving patches (21). Neuroevolution is proposed to develop deep attention convolutional neural networks for 2D and 3D medical image segmentation (22). A spatially weighted 3D network is proposed for single-modality segmentation of MRI brain tissue and extended it using multi-modality MRI data (23). 3D convolutional neural network based on 3D UNet and used rendering methods in computer graphics is proposed for 3D medical image segmentation (24). an Attention VNet module that uses 3D Attention Gate modules is proposed and applied to a semi-supervised learning-based left atrium segmentation framework (25).

An advanced deep learning network is proposed for 3D medical image segmentation (26). 3D ASPP module is proposed with a 3D DenseNet network (27). A new 3D medical image segmentation algorithm is proposed that defines the 3D brain tumor semantic segmentation task as a sequence-to-sequence prediction challenge in their study (28). An improved network based on 3D UNet is proposed to address the problem of low segmentation accuracy in the original 3D UNet network (29). Autopath, a more efficient image-specific inference method is proposed for 3D segmentation (30).

The method is proposed to achieve competitive accuracy from “weakly annotated" images, where weak annotations are obtained by representing the 3D boundary of the object of interest (31). A reinforcement learning-based 3D multimodal medical image segmentation algorithm is proposed with big data analysis (32). An interactive image segmentation tool is proposed that provides effective segmentation with multiple labels for both 2D and 3D medical images (33). A multi-agent approach for 3D medical image segmentation based on a group of autonomous interactive agents is proposed (34). The development of 3D network structures and the possibility of developing 3D networks is proposed for volume segmentation using 2D neural networks (35).

## 3. Methods

### 3.1. Automatic 3D segmentation framework of hyperintensities

**Figure 1:**
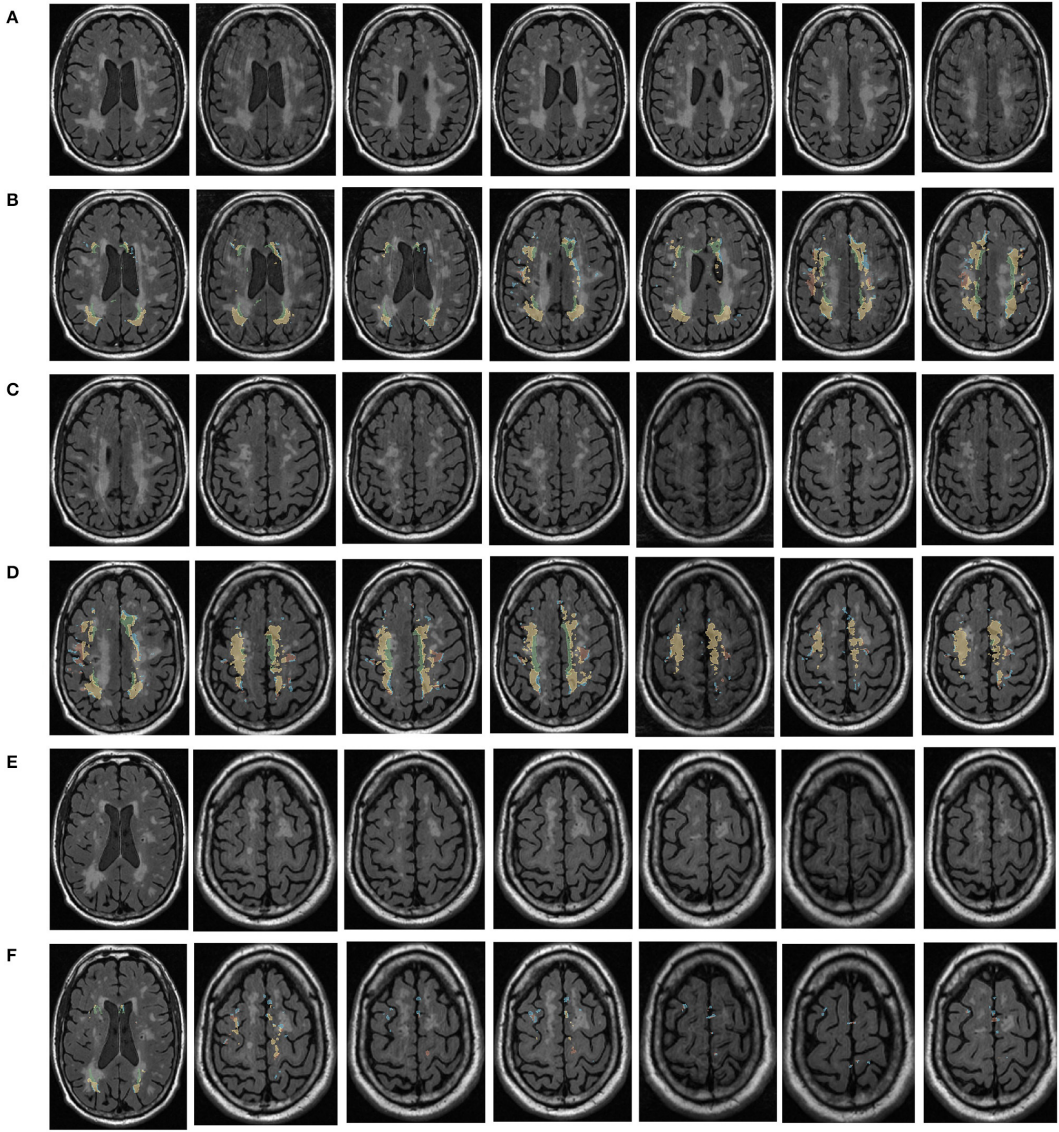
Visualization of white matter hyperintensity coverage. Rows **(A, C, E)** represent MRI visualizations of the brain not covered by cerebral white matter hyperintensity, rows **(B, D, F)** represent MRI images of cerebral white matter hyperintensity in brain regions that have produced a certain regional volume.

**Figure 2:**
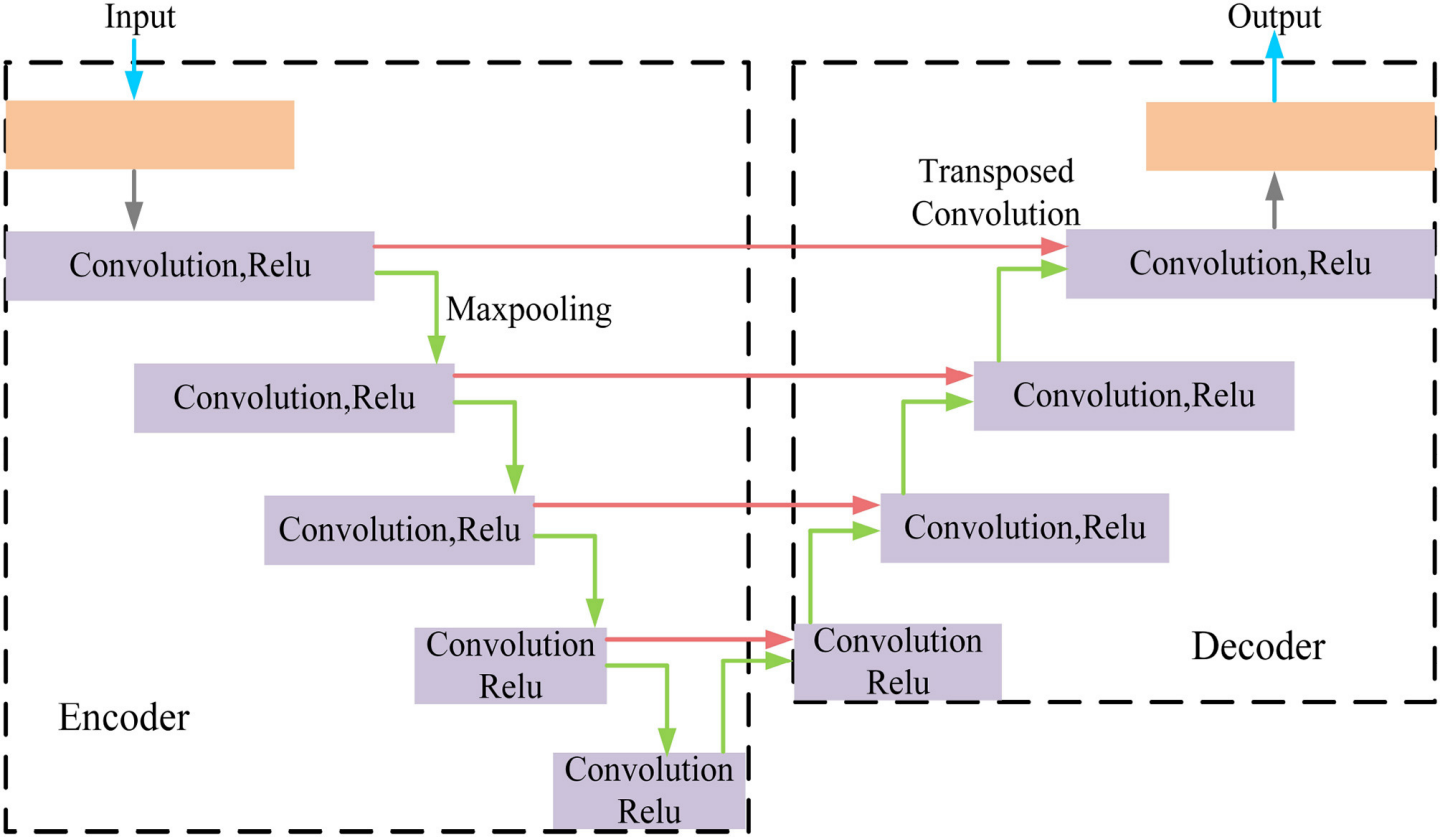
Deep encoder decoder module.

**Figure 3:**
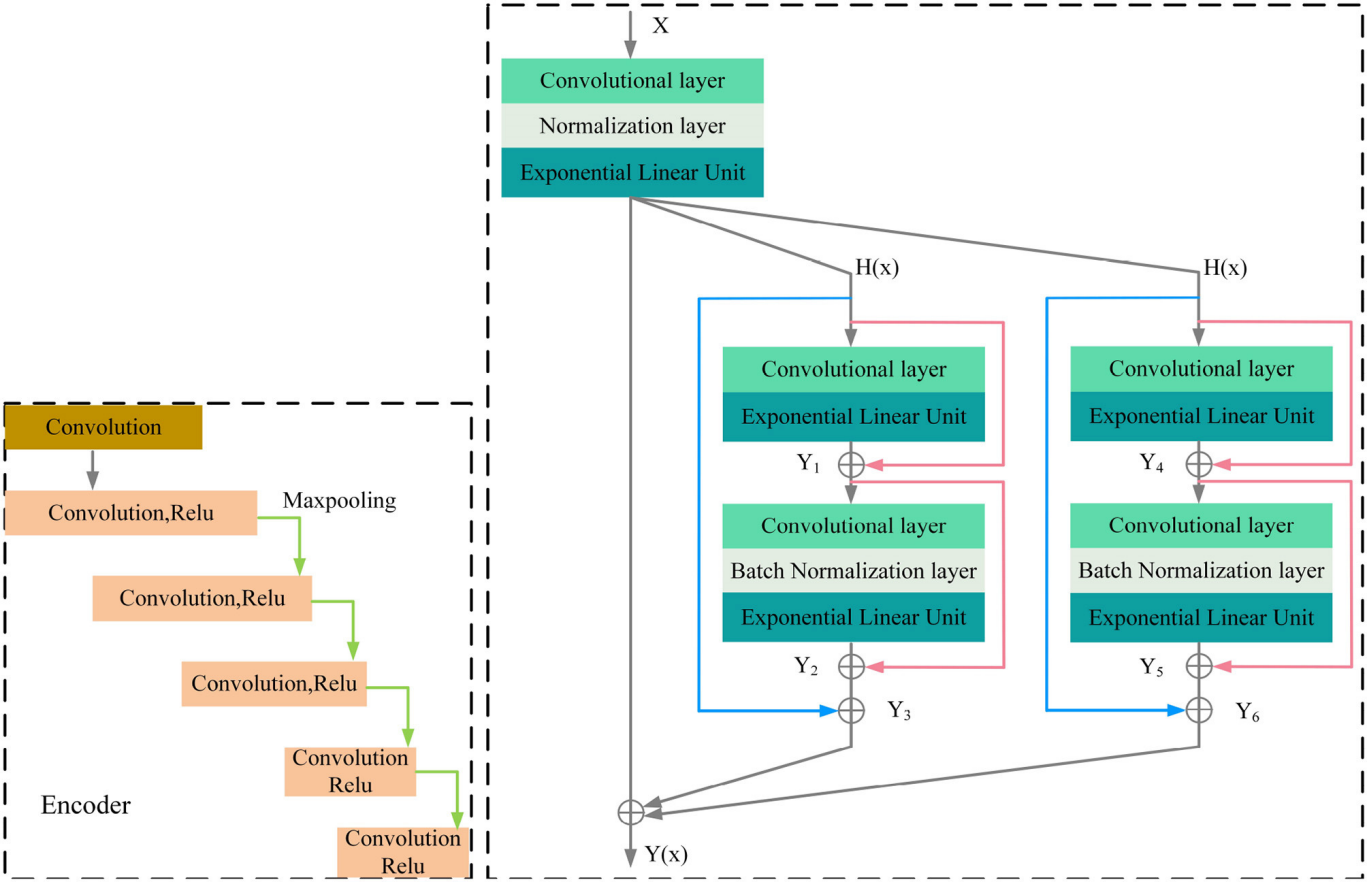
Multi-layer cross-connected residual mapping module.

**Figure 4:**
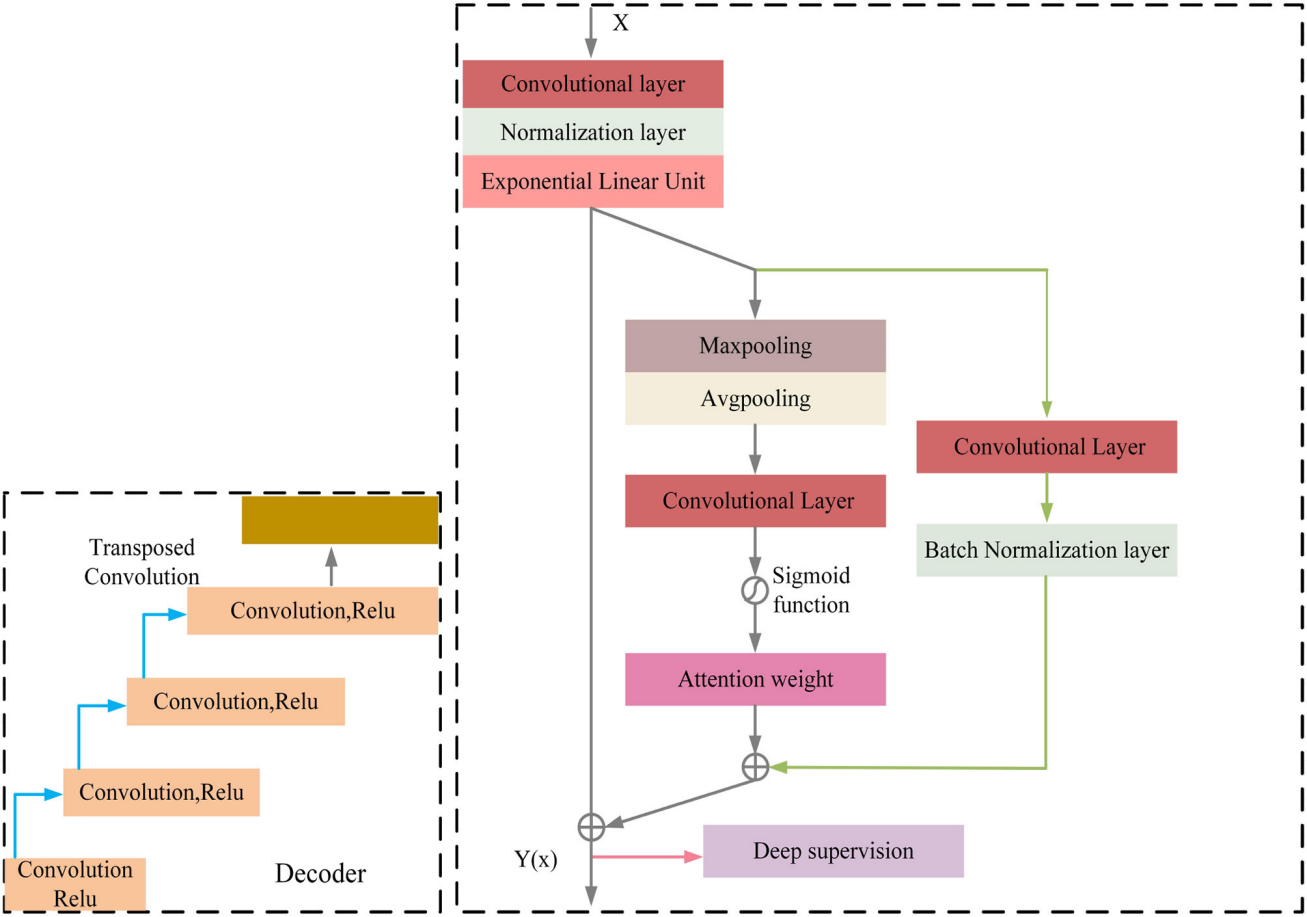
Spatial attention weighted enhanced supervision module.

MRI was performed with a 3T MRI scanner (Prisma, Siemens) using a 64-channel head coil. T2-fluid attenuated inversion recovery (FLAIR) images were acquired with the following parameters: TE = 150 ms, TR = 9,075 ms, TI = 2,250 ms, FOV = 256–256 mm^2^, matrix = 128–128, slice thickness = 2 mm, number of slices = 66. The visualization of the hyperintensities in the white matter of the brain is shown in Figure 1. In this paper, we propose a deep encoder-decoder module for 3D segmentation of brain white matter, as illustrated in Figure 2. Multi-layer Cross-connected Residual Mapping Module is proposed in the encoder stage to propose a three-dimensional multi-layer residual connectivity structure, as shown in Figure 3. In the decoder stage, Spatial Attention Weighted Enhanced Supervision Module is proposed, which proposes to enhance the depth supervision mechanism by the three-dimensional spatial attention mechanism, as shown in Figure 4.

#### 3.1.1. Multi-layer cross-connected residual mapping module

The three-dimensional residual structure with multi-level cross-connected proposed in the encoder stage can deepen the extraction of network ability for three-dimensional features by introducing the residual structure, which can effectively alleviate the problems such as gradient disappearance because of the network depth problem and adjust the weights of the previous neural network layers by learning the residual mapping function, the network can better adjust the input and output relationships of three-dimensional features. It is proposed that adding multi-level cross-connected to the three-dimensional residual structure can improve the nonlinear learning ability of the network and enhance the information exchange between different layers. The multi-level three-dimensional residual cross-connected structure incorporates multiple residual blocks and cross-connected structures in the encoder stage to achieve three-dimensional feature extraction and information exchange between the multi-level structures of the three-dimensional network. As is shown in Figure 3.

The encoder of the proposed model consists of multiple convolutional blocks, each of which followed by batch normalization and ELU activation. The cross-level residual connections are introduced by adding shortcut connections between the different levels of feature maps in the encoder. The cross-level residual connections enable information flow across different levels of feature maps, which helps to capture both low-level and high-level features for more accurate segmentation. This is achieved by adding the output of the previous block to the input of the current block, which allows the model to learn the residual features that are not captured by the previous block.

Regarding the calculation formulas of the Cross-Level Residual Connections Module, as shown in the Appendix, there are formula (1), formula (2), formula (3), formula (4), formula (5), formula (6), formula (7). In these formulas, *y* represents the computation result of each group convolution block, α and γ represent the coefficients of the cross-level connections, *K* represents the convolution kernels of the blocks, *b* represents the normalization coefficients, *Elu* represents the activation function.

#### 3.1.2. Spatial attention weighted enhanced supervision module

The module of deep supervision mechanism with spatial attention enhancement is proposed in the decoder stage, the spatial attention enhancement supervision function is proposed in the deep 3D residual network, which makes the network pay more attention to the important information in the input sequence to improve the performance of the model in the feature conversion stage, the spatial attention makes the network pay more attention to the important regions of the 3D data to improve the network perception in the decoder stage, the deep supervision mechanism can better learn the mapping relationship between the input 3D data to enhance the robustness of the model and improve the robustness in generative tasks.

The decoder of the proposed model also consists of multiple convolutional blocks, each of which followed by batch normalization and ELU activation. The spatial attention module is incorporated in the decoder to provide additional guidance for the model to focus on the most informative regions of the feature maps. The spatial attention module is designed to assign different weights to different regions of the feature maps based on their importance for the segmentation task. The attention module takes the feature maps of the previous layer as input and produces a spatial attention map, which is multiplied element-wise with the feature maps to obtain the attended feature maps. The attended feature maps highlight the most informative regions of the feature maps, which can improve the accuracy and robustness of the segmentation results. The spatial attention-guided supervision is introduced by incorporating the attended feature maps into the loss function as an additional term. This term encourages the model to focus on the most informative regions of the feature maps during training and helps to reduce the influence of the irrelevant regions (Figure 4).

Regarding the calculation formulas of the Spatial Attention Guided Supervision module, as shown in the Appendix, there are formula (8), formula (9), formula (10), formula (11), formula (12). In these formulas, *I*_*s*_ represents the computation result of the spatial attention, *I*_*c*_ represents the result of the shallow skip mapping, *I* represents the result of the enhanced spatial attention, *L* represents the result of the deep supervision, *Z* represents the result of the enhanced spatial attention and shallow residual connection, *Relu* and *Elu* represent the activation functions.

### 3.2. Correlation analysis of white matter hyperintensity cognitive assessment

**Figure 5:**
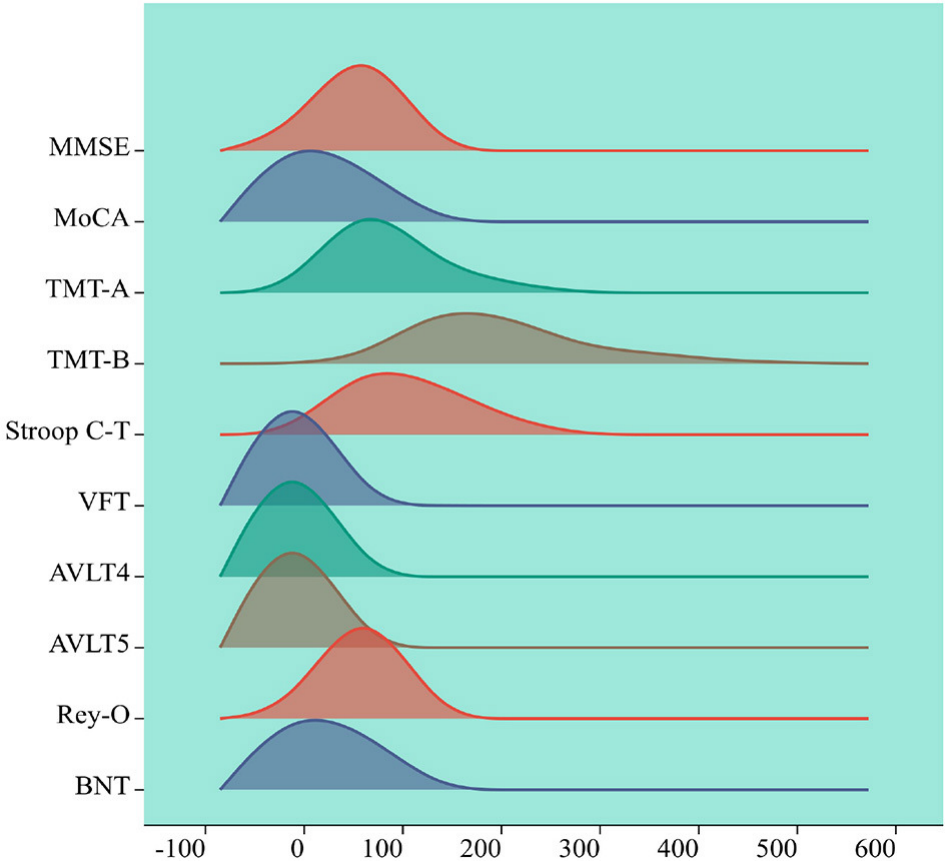
Visualization of cognitive assessment factors. The horizontal axis represents the range of values for the cognitive assessment coefficients, the vertical axis represents the density of cognitive assessment coefficient points within the range of values changed, each peak represents a peak in the cognitive assessment coefficient, the height of the peak represents the cognitive assessment coefficient density.

Cognitive function assessment coefficients are measures of people's cognitive abilities, including attention, memory, language, and spatial expression. Correlation analysis and multiple comparison analysis of cognitive function coefficients related to brain white matter can reveal the relationship between these indicators. Correlation analysis is a method to measure the strength of the relationship used for variables, usually using positive or negative correlation to determine the correlation, multiple comparison is a method used for differences between multiple variables. This paper uses SPSS software to conduct correlation analysis and multiple comparisons of cognitive coefficients in three cognitive states. The cognitive function coefficients involved in this paper are MMSE, MoCA, TMT-A, TMT-B, Stroop C-T, VFT, AVLT4, AVLT5, Rey-O, and BNT, with gender, age and education as covariates. Figure 5 summarizes the statistical data and factors related to cerebrovascular diseases.

The cognitive function assessment coefficients and their meanings in the statistics of this paper are as follows, MMSE: Mini-Mental State Examination, a standardized measurement tool to assess cognitive function, is commonly used to test the cognitive function of older adults. MoCA: Montreal Cognitive Assessment, a standardized measure of cognitive function, is also commonly used to test cognitive function in older adults. TMT-A and TMT-B: Trail Making Test A and B, a standardized measure to assess cognitive function, is also commonly used to test cognitive flexibility and executive function. TMT-A test requires subjects to connect numbers and TMT-B test requires subjects to connect numbers and letters alternately. Stroop C-T: The Stroop Color-Word Test, a standardized measure of cognitive function and inhibitory control, is a C-T task that requires subjects to quickly identify colors and inhibit word information that contradicts them. The VFT: Verbal Fluency Test, a standardized measure that assesses cognitive functioning and language ability, consists of two tasks: semantic category and letter category. AVLT4 and AVLT5: California Verbal Learning Test 4th and 5th Trials, a standardized measure to assess memory function. Rey-O: It represents Complex Figure Test, a standardized measure assessing visuospatial and memory functions. The BNT: Boston Naming Test, a standardized measure of verbal and lexical ability.

This study visualized cognitive assessment parameters associated with brain white matter hyperintensity while considering gender, age and education level as covariates. The remaining 10 cognitive function coefficients were analyzed and presented, providing a clearer understanding of the distribution of cognitive assessment parameters among subjects. This information can serve as a reference for subsequent analysis and decision making regarding cognitive assessment parameters closely related to brain white matter hyperintensity. The cognitive assessment coefficients corresponding to the white matter high-signal dataset were first visualized with gender, age and education as covariates among the subjects, the remaining 10 variables were visualized with numerical density. As is shown in Figure 5.

## 4. Result

**Figure 6:**
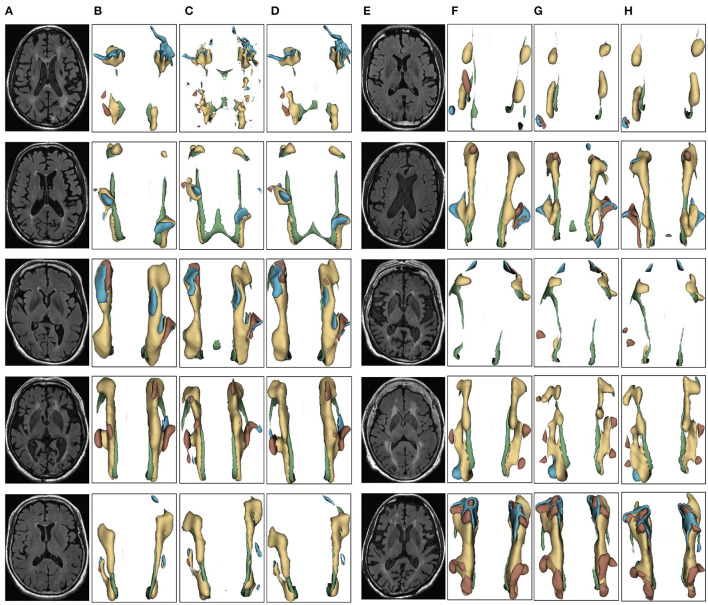
Segmentation results of three-dimensional white matter hyperintensity of Dementia. Columns **(A, E)** represent the white matter MRI of the brain ready for inference, columns **(B, F)** represent the brain white matter hyperintensity results of nnunet segmentation, columns **(C, G)** represent the brain white matter hyperintensity results of nnunet-resnet segmentation, columns **(D, H)** represent the brain white matter hyperintensity segmentation results of the automatic 3D segmentation framework proposed in this paper. The volumes of the brain white matter hyperintensity volume corresponding to Dementia obtained under the three segmentation algorithms are shown in Table 3. It can be seen that the brain white matter hyperintensity volume obtained by nnunet and nnunet-resnet segmentation has incomplete segmentation and over-segmentation, the volume obtained by the algorithm proposed in this paper is closest to the original brain white matter hyperintensity volume.

**Figure 7:**
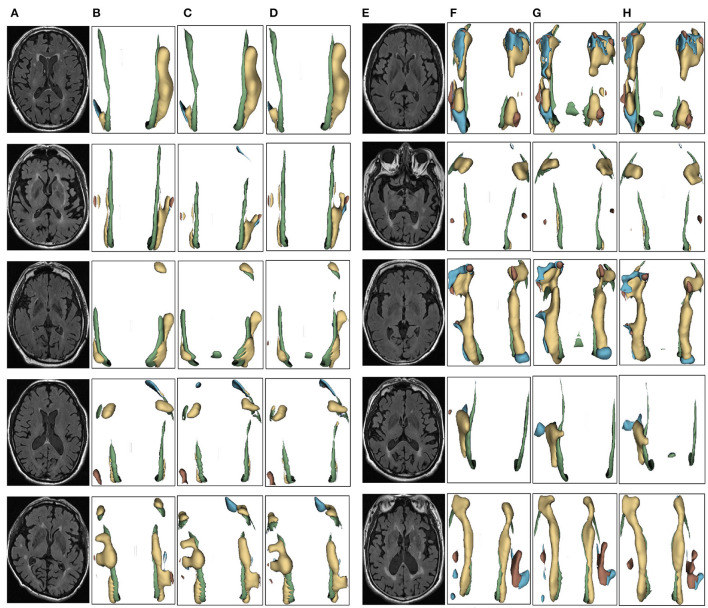
Segmentation results of three-dimensional white matter hyperintensity of MCI. Columns **(A, E)** represent the white matter MRI of the brain ready for inference, columns **(B, F)** represent the brain white matter hyperintensity results of nnunet segmentation, columns **(C, G)** represent the brain white matter hyperintensity results of nnunet-resnet segmentation, columns **(D, H)** represent the brain white matter hyperintensity segmentation results of the automatic 3D segmentation framework proposed in this paper. The volumes of the brain white matter hyperintensity volume corresponding to MCI obtained under the three segmentation algorithms are shown in Table 4. It can be seen that the brain white matter hyperintensity volume obtained by nnunet and nnunet-resnet segmentation has incomplete segmentation and over-segmentation, the volume obtained by the algorithm proposed in this paper is closest to the original brain white matter hyperintensity volume.

**Figure 8:**
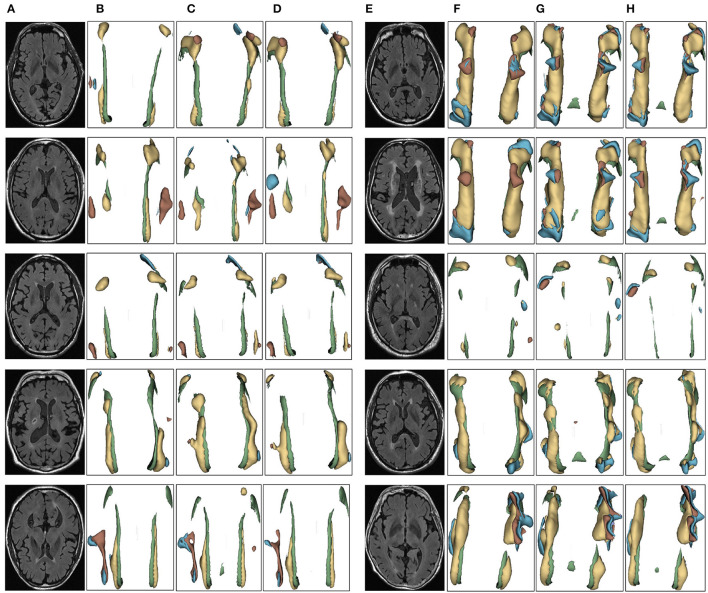
Segmentation results of three-dimensional white matter hyperintensity of NCI. Columns **(A, E)** represent the white matter MRI of the brain ready for inference, columns **(B, F)** represent the brain white matter hyperintensity results of nnunet segmentation, columns **(C, G)** represent the brain white matter hyperintensity results of nnunet-resnet segmentation, columns **(D, H)** represent the brain white matter hyperintensity segmentation results of the automatic 3D segmentation framework proposed in this paper. The volumes of the brain white matter hyperintensity volume corresponding to NCI obtained under the three segmentation algorithms are shown in Table 5. It can be seen that the brain white matter hyperintensity volume obtained by nnunet and nnunet-resnet segmentation has incomplete segmentation and over-segmentation, the volume obtained by the algorithm proposed in this paper is closest to the original brain white matter hyperintensity volume.

We evaluated the proposed the automatic 3D segmentation framework on a large public dataset for brain white matter hyperintensity segmentation, which contains MRI scans from patients with Alzheimer's disease, cognitive impairment and normal cognition. The dataset consists of 254 FLAIR images with manual annotations of brain white matter regions. Our experiments showed that the proposed the automatic 3D segmentation framework with cross-level residual connections in the encoder and spatial attention-guided supervision in the decoder achieved state of the art performance in terms of Segmentation accuracy. This dataset contains three kinds of data with different cognitive functions, dementia, MCI, NCI. The three automatic segmentation framework proposed in this paper is used to segment each of the three datasets. The segmentation results of dementia are shown in Figure 6, the segmentation results of MCI are shown in Figure 7, the segmentation results of NCI are shown in Figure 8.

The above standardized measurement tools and tests can assess multiple domains of cognitive function, including attention, memory, orientation, computational skills, language skills, executive function and visuospatial abilities. In clinical and research settings, these tools can be used to assess impairment or improvement in cognitive function, diagnose cognitive dysfunction or neurodegenerative disorders and develop individualized rehabilitation plans and treatment protocols.

In conducting the correlation experiment part, the correlation analysis was first performed on the cognitive function score coefficients related to cerebral white matter hyperintensity. The gender, age and education level of the subjects were used as covariates in the correlation analysis. In terms of gender, women would have better performance results than men on some cognitive assessments, age would also affect the decline in cognitive function, the level of education would also affect cognitive ability, these three factors would interfere with the study results and affect the accurate assessment of the relationship between cerebral white matter hyperintensity and cognitive function.

After the correlation analysis of the cognitive assessment coefficients, the results are presented as demonstrated in Table 1, from which it can be seen that the calculated *p*-values for MMSE and MoCA are <0.001, which indicates a significant association between the volume of white matter hyperintensity regions and cognitive function, *p*-values corresponding to these two cognitive assessment coefficients are <0.001 implying that the probability of observing a correlation as strong as the one found by chance in the study is <0.1%.

**Table 1:** Demographics and test performance of the study.

**Variable**	**Controls^a^**	**Patients with MCI^a^**	**Patients with dementia^a^**	***P*-value^b^**
*N*	44	82	8	N/A
Sex (female)	7	15	6	0.006
Age	75.875 ± 5.988	67.127 ± 7.205	65.75 ± 7.574	<0.001
Education	8.5 ± 4.242	10.178 ± 7.205	11.636 ± 2.805	<0.001
Mini-Mental State Examination (MMSE)	20.25 ± 3.767	27.165 ± 1.945	28.818 ± 1.093	<0.001
Montreal Cognitive Assessment (MoCA)	14.25 ± 2.817	21.608 ± 3.328	25.773 ± 2.575	<0.001
Trail making A (TMT-A)	145.5 ± 56.622	93.063 ± 44.844	61.432 ± 23.903	<0.001
Trail making B (TMT-B)	319 ± 72.856	223.101 ± 80.342	147.296 ± 37.690	<0.001
Stroop C-T	143.875 ± 38.908	121 ± 44.727	80.068 ± 15.623	<0.001
Verbal Fluency Test (VFT)	9.625 ± 1.867	13.747 ± 4.211	16.955 ± 3.219	<0.001
Auditory Verbal Learning Test4 (AVLT4)	1.75 ± 1.561	3.848 ± 2.129	6.773 ± 1.941	<0.001
Auditory Verbal Learning Test5 (AVLT5)	0.625 ± 0.857	2.848 ± 2.159	6.477 ± 2.169	<0.001
Rey-O	24.5 ± 5.523	32.911 ± 4.032	34.341 ± 3.819	<0.001
Boston Naming Test (BNT)	17.5 ± 4.770	22.532 ± 3.697	25.386 ± 2.90	<0.001

Correlation analysis of the cognitive function coefficients associated with high brain white matter signals showed that the *p*-value of all the function coefficients were <0.001, which indicated that the statistical results were significant.

^a^Mean ± standard deviation (SD).

^b^*p*-values from three-sample *t*-tests between the three groups.

The probability of observing a correlation as strong as the one found by chance in the study is <0.1%. That is the greater the white matter region of the brain in the subject, the smaller the corresponding MMSE and MoCA values, the two showed a negative correlation, suggesting an association between cognitive decline, neurodegenerative disorders and white matter hyperintensity, which clinically suggests the need for further examination and treatment, identifying which cognitive functions are affected in the patient, a more targeted approach to rehabilitation can be developed treatment approach. In the 3D segmentation experiments of three groups of brain white matter hyperintensity, the segmentation results of brain white matter hyperintensity obtained by training prediction of the automatic 3D segmentation framework proposed in this paper are shown in Figures 6–8. nnunet and nnunet-resnet is selected as the control experiment for the segmentation results, which is also shown in Figures 6–8.

**Figure 9:**
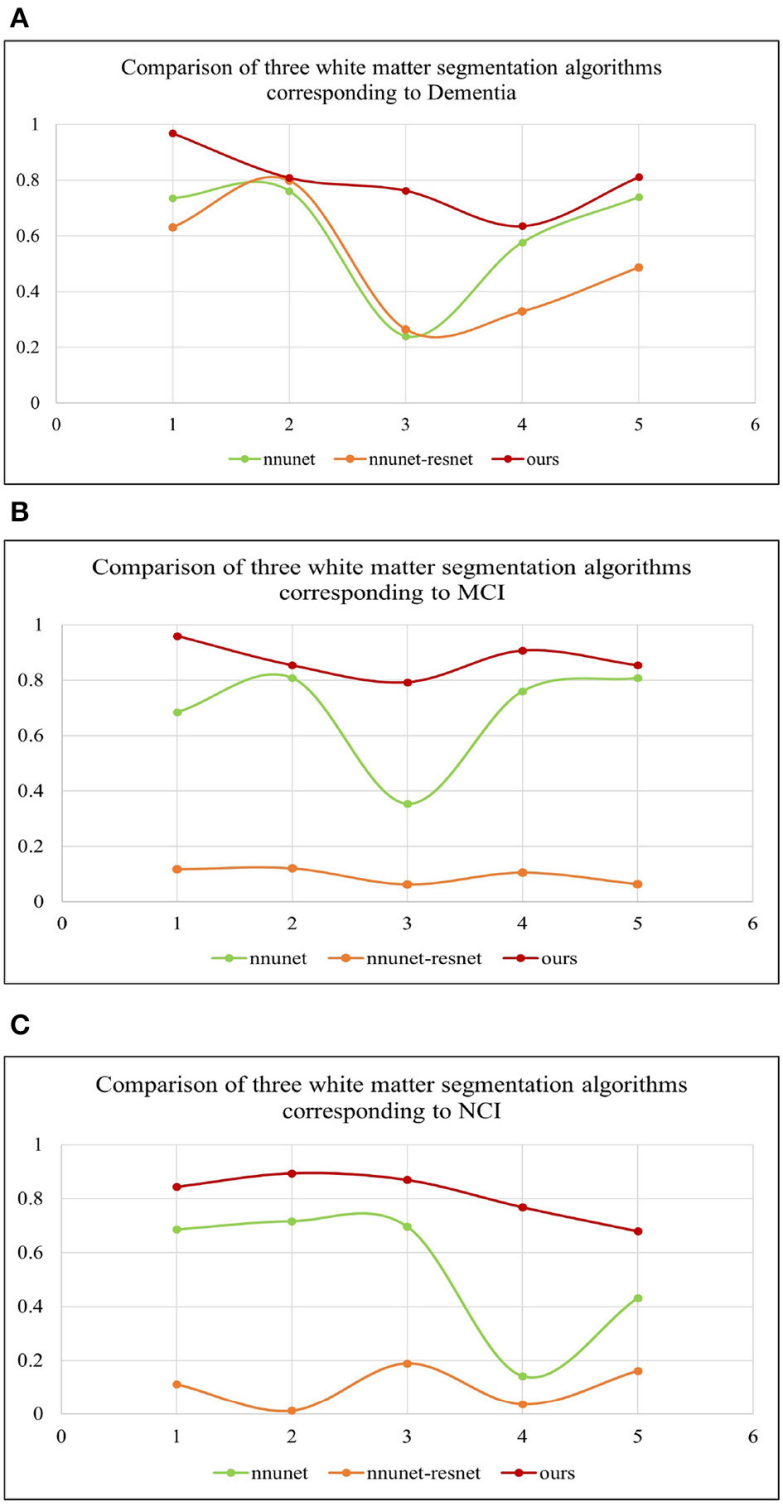
**(A–C)** Represent three different states of white matter hyperintensity. Dementia, MCI and NCI, corresponding to the comparison of white matter volume obtained by the three segmentation algorithms.

**Figure 10:**
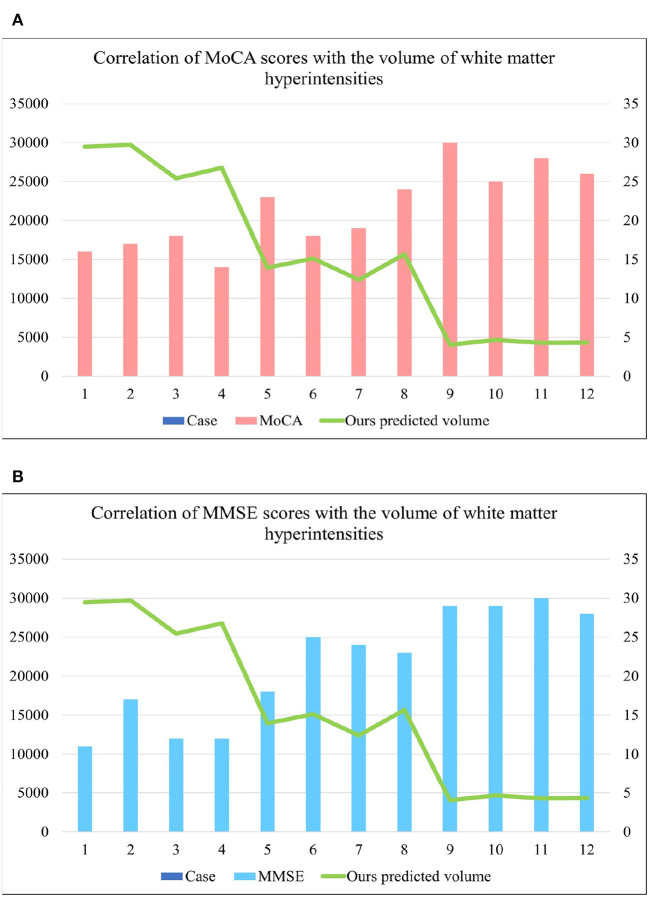
**(A)** Represents the correlation between MoCA score and cerebral white matter hyperintensity volume, from which it is known that there is a negative correlation between MoCA and cerebral white matter hyperintensity volume. **(B)** Represents the correlation between MMSE score and cerebral white matter hyperintensity volume, from which it is known that there is a negative correlation between MMSE and cerebral white matter hyperintensity volume. where cases 1–4 are the values of brain white matter hyperintensity volume, MoCA and MMSE corresponding to dementia, case 5–8 are the values of brain white matter hyperintensity volume, MoCA and MMSE corresponding to MCI, cases 9–12 are the values of brainwhite matter hyperintensity volume, MoCA and MMSE corresponding to NCI.

Table 2 shows the multiple comparisons between the relevant influencing factors for Dementia, NCI and MCI. It shows the significance between the relevant factors. Figure 9 represents the accuracy ratio of brain white matter volume obtained by the three segmentation algorithms, it can be seen that the automatic brain white matter 3D segmentation algorithm proposed in this paper outperforms the other two segmentation algorithms in terms of the accuracy of brain white matter segmentation in Demntia, NCI and MCI. The horizontal axis represents the number of experiments, the vertical axis represents the white matter volume accuracy ratio obtained by algorithm segmentation. The correlation between cerebral white matter hyperintensity volume, MoCA and MMSE scores is shown in Figure 10, from which it can be seen that there is a negative correlation between cerebral white matter hyperintensity volume and MoCA and MMSE scores, as the volume of the three cognitive states dementia, MCI, and NCI corresponding to cerebral white matter hyperintensity decreases in the state, the values of MoCA and MMSE subsequently increase.

**Table 2:** Multiple comparisons between factors related to Dementia, MCI and NCI, ^*^the significance level of the difference between the average values is 0.05.

**Factors**		**(I)Sample**	**(J)Sample**	**Mean difference(I-J)**	**Standard error**	**Significance**
MMSE	LSD	1	2	−6.915^*^	0.707	<0.001
			3	−8.568^*^	0.733	<0.001
		2	1	6.915^*^	0.707	<0.001
			3	−1.654^*^	0.359	<0.001
		3	1	8.568^*^	0.733	<0.001
			2	1.654^*^	0.359	<0.001
MoCA	LSD	1	2	−7.358^*^	1.150	<0.001
			3	−11.523^*^	1.191	<0.001
		2	1	7.358^*^	1.150	<0.001
			3	−4.165^*^	0.583	<0.001
		3	1	11.523^*^	1.191	<0.001
			2	4.165^*^	0.583	<0.001
TMT-A	LSD	1	2	52.437^*^	15.016	<0.001
			3	84.068^*^	15.555	<0.001
		2	1	−52.437^*^	15.016	<0.001
			3	31.631^*^	7.613	<0.001
		3	1	−84.068^*^	15.555	<0.001
			2	−31.631^*^	7.613	<0.001
TMT-B	LSD	1	2	95.899^*^	25.716	<0.001
			3	171.705^*^	26.640	<0.001
		2	1	−95.899^*^	25.716	<0.001
			3	75.806^*^	13.038	<0.001
		3	1	−171.705^*^	26.640	<0.001
			2	−75.806^*^	13.038	<0.001
Stroop C-T	LSD	1	2	22.875	13.948	0.103
			3	63.807^*^	14.449	<0.001
		2	1	−22.875	13.948	0.103
			3	40.932^*^	7.072	<0.001
		3	1	−63.807^*^	14.449	<0.001
			2	−40.932^*^	7.072	<0.001
VFT	LSD	1	2	−4.122^*^	1.424	0.004
			3	−7.330^*^	1.475	<0.001
		2	1	4.122^*^	1.424	0.004
			3	−3.208^*^	0.722	<0.001
		3	1	7.330^*^	1.475	<0.001
			2	3.208^*^	0.722	<0.001
AVLT4	LSD	1	2	−2.098^*^	0.764	0.007
			3	−5.023^*^	0.792	<0.001
		2	1	2.098^*^	0.764	0.007
			3	−2.925^*^	0.388	<0.001
		3	1	5.023^*^	0.792	<0.001
			2	2.925^*^	0.388	<0.001
AVLT5	LSD	1	2	−2.223^*^	0.790	0.006
			3	−5.852^*^	0.819	<0.001
		2	1	2.223^*^	0.790	0.006
			3	−3.629^*^	0.401	<0.001
		3	1	5.852^*^	0.819	<0.001
			2	3.629^*^	0.401	<0.001
Rey-O	LSD	1	2	−8.411^*^	1.528	<0.001
			3	−9.841^*^	1.583	<0.001
		2	1	8.411^*^	1.528	<0.001
			3	−1.430	0.775	0.067
		3	1	9.841^*^	1.583	<0.001
			2	1.430	0.775	<0.001
BNT	LSD	1	2	−5.032^*^	1.313	<0.001
			3	−7.886^*^	1.361	<0.001
		2	1	5.032^*^	1.313	<0.001
			3	−2.855^*^	0.666	<0.001
		3	1	7.886^*^	1.361	<0.001
			2	2.855^*^	0.666	<0.001

We propose an automatic 3D segmentation framework for white matter hyperintensity using a deep multi-mapping encoder-decoder structure. The segmentation method proposed in this paper was compared with nnunet and nnunet-resnet for 3D segmentation volume of cerebral white matter as demonstrated in Tables 3–5. The first column indicates the subject's serial number, the second column indicates the volume of the subject's original brain white matter hyperintensity, the third column indicates the volume of the brain white matter hyperintensity obtained after the nnunet predictive inference segmentation, the fourth column indicates the volume of the brain white matter hyperintensity obtained after the nnunet-resnet predictive inference segmentation, the fifth column indicates the volume of the brain white matter hyperintensity obtained after the predictive inference segmentation by the automatic 3D segmentation framework proposed in this paper, the sixth column represents the ratio of the volume of the brain white matter hyperintensity obtained by nnunet segmentation corresponding to the original corresponding brain white matter hyperintensity volume, the seventh column represents the ratio of the volume of the brain white matter hyperintensity obtained by nnunet-resnet segmentation corresponding to the original corresponding brain white matter hyperintensity volume, the eighth column represents the ratio of the volume of the brain white matter hyperintensity obtained by the automatic 3D segmentation framework segmented in this paper corresponding to the original corresponding brain white matter hyperintensity volume. The values of the ratio corresponding to the sixth, seventh and eighth columns, a positive value means that the prediction of segmentation is correct and the closer to 1, the more accurate the segmentation is, a negative value indicates an over-prediction, indicating that there is an over-segmentation in the 3D segmentation process, which is a wrong prediction.

**Table 3:** Comparative analysis of white matter segmentation volumes corresponding to Dementia obtained by three segmentation algorithms.

**Dementia case**	**Original (mm^3^)**	**nnunet (mm^3^)**	**nnunet-resnet (mm^3^)**	**Ours (mm^3^)**	**Ratio^b^**	**Ratio^c^**	**Ratio^d^**
1	29,836	25,752	29,534	**29,474**	0.8631	0.9898	**0.9879**
2	11,298.1	8,600	9,018	**9,138**	0.7611	0.7981	**0.8088**
3	31,893.6	1,452.1	5,728.4	**29,711**	0.0455	0.1796	**0.9316**
4	32,734	2,088	1,902	**4,108**	0.0637	0.0581	**0.1255**
5	55,380	7,993.6	3,022.6	**53,673**	0.1443	0.0545	**0.9692**
	32,228.34^a^	9,177.14^a^	9,841^a^	**25,220.8** **^a^**		
6	54,510	1,832	1,834	**4,108**	0.0336	0.0336	**0.0754**
7	68,029.6	27,628.7	27,946.7	**34,154**	0.4061	0.4108	**0.5020**
8	28,806.4	1,500.1	1,372.1	**25,422**	0.0520	0.0476	**0.8825**
9	18,753	32,543.8	30,587.7	**18,160**	−0.7353	−0.6310	**0.9684**
10	9,592	18,328	17,842	**3,014**	−0.9107	−0.8600	**0.3142**
	35,938.2^a^	16,366.52^a^	15,916.5^a^	**16,971.6** **^a^**		
11	48,481.4	700	564	**7,286**	0.0144	0.0116	**0.1503**
12	42,134	24,284.6	13,850.6	**26,770**	0.5763	0.3287	**0.6354**
13	26,460	716	14	**1,694**	0.0270	0.0005	**0.0640**
14	3,329.6	4,536	4,244	**1,836**	−0.3623	−0.2746	**0.5514**
15	20,715.2	25,690	26,182	**15,796**	−0.2401	−0.2639	**0.7625**
	28,224.04^a^	11,185.32^a^	8,970.92^a^	**10,676.4** ^***a***^		
16	43,534.4	8,674	9,068	**29,011.5**	0.1992	0.2082	**0.6664**
17	4,294	764.2	0	**3,704**	0.1779	0	**0.8626**
18	4,124	6,052	14,948	**2,430**	−0.4675	−0.6246	**0.5892**
19	35,618	670	552	**27,478**	0.0188	0.0155	**0.7715**
20	22,394	56,541.6	55,677.5	**18,160**	0.7386	−0.4862	**0.8109**
	21,992.88^a^	6,540.36^a^	16,049.1^a^	**16,156.7** **^a^**		

In the 3D segmentation of the brain white matter hyperintensity of Dementia, nnunet and nnunet-resnet were selected as the comparison test. The closer the segmentation ratio is to 1, the higher the segmentation accuracy is, a negative value indicated over-segmentation. From the final segmentation volume, the segmentation results of nnunet and nnunet-resnet are under-segmented and over-segmented, while the segmentation results of the automatic 3D framework proposed in this paper are more accurate. ^*a*^Mean. ^*b*^Ratio of nnunet. ^*c*^Ratio of nnunet-resnet. ^*d*^Ratio of ours. The bold values indicate that the evaluation value obtained by the segmentation method proposed in this study is better than other 3D segmentation methods.

**Table 4:** Comparative analysis of white matter segmentation volumes corresponding to MCI obtained by three segmentation algorithms.

**MCI case**	**Original (mm^3^)**	**nnunet (mm^3^)**	**nnunet-resnet (mm^3^)**	**Ours (mm^3^)**	**Ratio^b^**	**Ratio^c^**	**Ratio^d^**
1	14,214	9,728	440	**13,958**	0.6843	0.0309	**0.9820**
2	12,562	4,436	376	**9,954**	0.3531	0.0299	**0.7924**
3	17,130	6,818	1,064	**15,122**	0.3980	0.0621	**0.8828**
4	12,924	6,442	804	**12,404**	0.4984	0.0622	**0.9598**
5	17,508	5,866	1,290	**14,958**	0.3350	0.0736	**0.8544**
	14,867.6^a^	6,658^a^	794.8^a^	**13,279.2** **^a^**			
6	13,938	4,956	370	**7,440**	0.3555	0.0265	**0.5338**
7	18,816	4,968	4,450	**13,670**	0.2640	0.23655	**0.7265**
8	10,890	15,790	4	**9,326**	−0.4499	0.0004	**0.8564**
9	13,098	9,956	1,376	**11,880**	0.7601	0.1051	**0.9070**
10	9,105.2	10,314	1,070	**7,668**	−0.1327	0.1175	**0.8422**
	13,169.44^a^	9,196.8^a^	1,454^a^	**9,996.8** **^a^**			
11	12,898	17,592	168	**7,966**	−0.3639	0.0130	**0.6176**
12	9,948	8,222.9	0	**9,638**	0.8265	0	**0.9688**
13	17,863	9,962	116	**15,678**	0.5576	0.0065	**0.8777**
14	12,530.7	10,130	796	**10,700**	0.8084	0.0635	**0.8539**
15	15,440	18,420	2,076	**12,826**	−0.1930	0.1345	**0.8307**
	13,735.94^a^	12,865.38^a^	631.2^a^	**11,361.6** **^a^**			
16	14,580	4,648.8	1,038	**5,630**	0.3188	0.0712	**0.3861**
17	15,232	9,068.8	188	**12,634**	0.5953	0.0123	**0.8294**
18	10,056	15,858	1,212	**7,599**	−0.5769	0.1205	**0.7557**
19	9,858	5,000	3,864	**5,194**	0.5072	0.3920	**0.5269**
20	16,501	8,704	1,440	**16,160**	0.5274	0.0873	**0.9793**
	13,245.4^a^	8,655.92^a^	1,548.4^a^	**9,443.4** **^a^**			

In the 3D segmentation of the brain white matter hyperintensity of MCI, nnunet and nnunet-resnet were selected as the comparison test. The closer the segmentation ratio is to 1, the higher the segmentation accuracy is, a negative value indicated over-segmentation. From the final segmentation volume, the segmentation results of nnunet and nnunet-resnet are under-segmented and over-segmented, while the segmentation results of the automatic 3D framework proposed in this paper are more accurate.

^a^Mean.

^b^Ratio of nnunet.

^c^Ratio of nnunet-resnet.

^d^Ratio of ours. The bold values indicate that the evaluation value obtained by the segmentation method proposed in this study is better than other 3D segmentation methods.

**Table 5:** Comparative analysis of white matter segmentation volumes corresponding to NCI obtained by three segmentation algorithms.

**NCI case**	**Original (mm^3^)**	**nnunet (mm^3^)**	**nnunet-resnet (mm^3^)**	**Ours (mm^3^)**	**Ratio^b^**	**Ratio^c^**	**Ratio^d^**
1	2,358	4,046	9,460	**2,108**	−0.7158	−0.0119	**0.8940**
2	1,806	624.1	4,732	**794**	0.3455	−0.6202	**0.4396**
3	1,790	990	12,142	**1,598**	0.5530	−0.7832	**0.8927**
4	5,615.4	676	10,722	**4,046**	0.1203	−0.9094	**0.7205**
5	2,994	3,150	9,976	**2,642**	−0.0521	−0.3320	**0.8824**
	2,912.68^a^	1,897.22^a^	9,406.4^a^	**2,237.6** **^a^**		
6	2,778	2,322	13,366	**1,966**	0.8358	−0.8114	**0.7077**
7	2,630.2	170	14,418	**566**	0.0646	−0.4817	**0.2152**
8	5,560	3,810	622	**4,692**	0.6852	0.1119	**0.8439**
9	4,056	574	32,594	**3,116**	0.1415	−0.0360	**0.7682**
10	4,118	2,606.2	11,934.8	**2,812**	0.6328	−0.8982	**0.6829**
	3,828.44^a^	1,896.44^a^	14,586.96^a^	**2,630.4** **^a^**		
11	6,338	2,732	32,712	**4,305**	0.4310	−0.1612	**0.6792**
12	1,934	1,144	624.2	**1,058**	0.5915	0.3228	**0.5471**
13	1,448	539.9	14,170	**468**	0.3728	−0.7859	**0.3232**
14	1,716	236	42	**348**	0.1375	0.02448	**0.2028**
15	3,064	240	33,756	**2,750**	0.7859	−0.0170	**0.8975**
	2,900^a^	1,411.98^a^	16,260.84^a^	**1,785.8** **^a^**		
16	5,426	1,074	6,189	**4,310**	0.1979	−0.1406	**0.7943**
17	3,009.4	202	8,694.8	**2,262**	0.0671	−0.8892	**0.7516**
18	3,958	1,434	14,660	**2,292**	0.3623	−0.7039	**0.5791**
19	2,216	3,328	4,870	**370**	−0.5018	−0.1977	**0.1670**
20	1,962	1,366	10,180	**1,706**	0.6962	−0.1889	**0.8695**
	3,314.28^a^	1,480.8^a^	8,918.76^a^	**2,188** **^a^**		

In the 3D segmentation of the brain white matter hyperintensity of NCI, nnunet and nnunet-resnet were selected as the comparison test. The closer the segmentation ratio is to 1, the higher the segmentation accuracy is, a negative value indicated over-segmentation. From the final segmentation volume, the segmentation results of nnunet and nnunet-resnet are under-segmented and over-segmented, while the segmentation results of the automatic 3D framework proposed in this paper are more accurate.

^a^Mean.

^b^Ratio of nnunet.

^c^Ratio of nnunet-resnet.

^d^Ratio of ours. The bold values indicate that the evaluation value obtained by the segmentation method proposed in this study is better than other 3D segmentation methods.

From the results in Tables 3–5, the predicted segmentation accuracy of the automatic 3D segmentation framework proposed in this paper is higher than that of nnunet and nnunet-resnet, the segmented volumes of brain white matter hyperintensity obtained from the predicted segmentation by the automatic 3D segmentation framework, the nnunet framework, the nnunet-resnet framework show that the volume of brain white matter hyperintensity regions of Dementia is larger than that of MCI. The volume of cerebral white matter hyperintensity area for MCI was larger than that for NCI. The difference in white matter hyperintensity volume between the three cognitive states can be used to assess the severity and progression of the disease and to develop a clinical treatment plan that meets individual needs.

## 5. Discussion

White matter hyperintensity is an abnormal signal in the white matter of the brain, clinically manifested as hyperintensity in MRI images. It is commonly associated with cerebral blood disease, small vessel disease and neurodegenerative disease. Accurate segmentation of these areas has greatly improved the treatment of cerebrovascular and small vessel diseases. Progress in the treatment of neurodegenerative diseases like dementia has also been made with the help of efficient segmentation techniques.

This paper proposes a 3D segmentation method for the white matter hyperintensity region using a 3D encoder and 3D decoder. The method incorporates a residual network into the global framework. While traditional medical image segmentation has been based on two dimensional images, this method utilizes three dimensional characteristics for improved accuracy. This study proposes the Multi-layer Cross-connected Residual Mapping Module in the decoder stage for enhancing the feature extraction and representation abilities of the segmentation model for MRI images. The module combines low-level and high-level feature information using a multi-layer cross-connected residual structure. The study introduces the Spatial Attention Weighted Enhanced Supervision Module in the decoder section, which utilizes the spatial attention mechanism to capture boundary information and regional small pixel information during 3D reconstruction. This enhances the supervisory role of the mechanism and improves the segmentation accuracy and reconstruction capability of the model.

This study analyzed the correlation of using cognitive assessment coefficients in the white matter hyperintensity segmentation experiment, taking into account the gender, age and education as covariates. The study then explored the correlation between the volume of the white matter hyperintensity area and the MMSE scores, MoCA scores. The results showed that a larger volume of the white matter hyperintensity was associated with lower MMSE and MoCA scores, indicating poorer cognitive function. This study conducted segmentation experiments on three different groups of white matter hyperintensity: Dementia, MCI, and NCI. By comparing the results of these experiments, the researchers found that the volume size of the white matter hyperintensity regions was largest in dementia, followed by MCI, NCI. The study also showed that the proposed three-dimensional segmentation framework had higher efficiency compared to other methods.

The proposed 3D segmentation framework in this paper has some limitations. The model design parameters are too large, which can lead to longer training and inference times. The segmentation algorithm requires high performance equipment to run effectively. In this paper, we propose that the limitations of the automatic 3D brain white matter hyperintensity segmentation framework exist for incomplete segmentation of small volume of brain white matter hyperintensity tissue, the correlation between the volume of brain white matter hyperintensity corresponding to the three cognitive states and more cognitive assessment parameters can be explored in the future, the correlation between the brain white matter hyperintensity segmentation work carried out with more cerebrovascular diseases and cognitive state disorders.

## 6. Conclusion

The contribution of this paper is to propose a three-dimensional automatic brain white matter hyperintensity volume segmentation framework, compared with nnunet and nnunet-resnet this proposed three-dimensional segmentation framework does not have incomplete segmentation and over-segmentation, this paper explored the correlation analysis between brain white matter hyperintensity volume and cognitive assessment coefficient MoCA, MMSE, and concluded that there is a negative correlation between the two.

The correlation analysis as well as multiple comparison analysis of the relevant cognitive functional coefficients affecting brain white matter was performed by SPSS. The results indicated that the *p*-value of each functional coefficient was <0.001, which indicated a negative correlation between white matter hyperintensity volume and MMSE and MoCA scores, which indicated that larger volumes of brain white matter hyperintensity volume correlated with lower functional coefficients, such as MMSE and MOCA. This thesis concludes that the segmentation comparison of three different groups of cerebral white matter hyperintensity volume between Dementia, MCI and NCI in descending order of Dementia, MCI and NCI. To verify that the automatic 3D framework proposed in this paper has a high efficiency, the comparison with nnunet and nnunet-resnet, the results obtained show that the segmentation volumes of nnunet and nnunet-resnet are under-segmented and over-segmented, while the results of the automatic three-dimensional segmentation framework proposed in this paper are more accurate than the other two methods, through the comparison of the segmentation volume rate and segmentation accuracy in the results, the automatic three-dimensional segmentation framework proposed in this paper has a higher. The results of the automatic three-dimensional segmentation framework proposed in this paper are more accurate than the other two methods.

## Data availability statement

The raw data supporting the conclusions of this article will be made available by the authors, without undue reservation.

## Ethics statement

The studies involving human participants were reviewed and approved by Shenzhen Second People's Hospital. The patients/participants provided their written informed consent to participate in this study.

## Author contributions

BX and XZ proposed methods and completed the whole experiment. WY and YW completed paper writing and revised the paper. DZ, CT, and XL completed the organization of the graphs and tables in the paper. XC made modifications to the overall arrangement of the paper. All authors contributed to the article and approved the submitted version.

## Appendix

(1)
$$\begin{array}{l} y_{1}=\alpha +X_{i}+Elu\overset{N}{\underset{i=1}{\sum }}\left(K_{c1}H\left(X_{i}\right)\right) \end{array}$$

(2)
$$\begin{array}{l} y_{2}=\alpha +y_{1}+Elu\overset{N}{\underset{i=1}{\sum }}\left(K_{c2}y_{1}+b_{2}\right) \end{array}$$

(3)
$$\begin{array}{l} y_{3}=\overset{N}{\underset{i=1}{\sum }}\left(H\left(X_{i}+\gamma y_{2}\right)\right) \end{array}$$

(4)
$$\begin{array}{l} y_{4}=\alpha +X_{i}+Elu\overset{N}{\underset{i=1}{\sum }}\left(K_{c3}H\left(X_{i}\right)\right) \end{array}$$

(5)
$$\begin{array}{l} y_{5}=\alpha +y_{4}+Elu\overset{N}{\underset{i=1}{\sum }}\left(K_{c4}y_{4}+b_{5}\right) \end{array}$$

(6)
$$\begin{array}{l} y_{6}=\overset{N}{\underset{i=1}{\sum }}\left(H\left(X_{i}+\gamma y_{5}\right)\right) \end{array}$$

(7)
$$\begin{array}{l} y=\overset{N}{\underset{i=1}{\sum }}\left(y_{3}+y_{6}+\left(KH\left(X_{i}\right)+b\right)\right) \end{array}$$

(8)
$$\begin{array}{l} I_{s}=Relu\left[F\left(Maxp\left(X\right),Avgp\left(X\right)\right)\right] \end{array}$$

(9)
$$\begin{array}{l} I_{c}=BN\left(K_{c}X\right) \end{array}$$

(10)
$$\begin{array}{l} I=I_{s}⊕I_{c} \end{array}$$

(11)
$$\begin{array}{l} Z=I⊕Elu\overset{N}{\underset{i=1}{\sum }}\left(KH\left(X_{i}\right)+b\right) \end{array}$$

(12)
$$\begin{array}{l} L=L_{el}\left(G,P\right) \end{array}$$

## References

[1] Li B Ohtomo R Thunemann M Adams SR Yang J Fu B. Two-photon microscopic imaging of capillary red blood cell flux in mouse brain reveals vulnerability of cerebral white matter to hypoperfusion. J Cereb Blood Flow Metab. (2020) 40:501–12. 10.1177/0271678X19831016 30829101 PMC7026840

[2] Cerri S Greve DN Hoopes A Lundell H Siebner HR Mühlau M. An open-source tool for longitudinal whole-brain and white matter lesion segmentation. NeuroImage Clin. (2023) 38:103354. 10.1016/j.nicl.2023.103354 36907041 PMC10024238

[3] Ding T Cohen A O'Connor E Karim H Crainiceanu A Muschelli J. An improved algorithm of white matter hyperintensity detection in elderly adults. NeuroImageClin. (2020) 25:102151. 10.1016/j.nicl.2019.102151 31927502 PMC6957792

[4] Alzaid H Ethofer T Hobert MA Kardatzki B Erb M Maetzler W. Distinct relationship between cognitive flexibility and white matter integrity in individuals at risk of Parkinson's disease. Front Aging Neurosci. (2020) 12:250. 10.3389/fnagi.2020.00250 32903902 PMC7439016

[5] Zhang W Zhou X Yin J Zhao W Huang C Zhang C. YKL-40 as a novel biomarker related to white matter damage and cognitive impairment in patients with cerebral small vessel disease. Brain Res. (2023) 1807:148318. 10.1016/j.brainres.2023.148318 36898474

[6] Andica C Kamagata K Aoki S. Automated three-dimensional major white matter bundle segmentation using diffusion magnetic resonance imaging. Anat Sci Int. (2023) 98:318–36. 10.1007/s12565-023-00715-9 37017902 PMC10256641

[7] Middlebrooks EH Lin C Westerhold E Okromelidze L Vibhute P Grewal SS. Improved detection of focal cortical dysplasia using a novel 3D imaging sequence: Edge-Enhancing Gradient Echo (3D-EDGE) MRI. NeuroImage Clin. (2020) 28:102449. 10.1016/j.nicl.2020.102449 33032066 PMC7552096

[8] Kaur A Kaur L Singh A. GA-UNet: UNet-based framework for segmentation of 2D and 3D medical images applicable on heterogeneous datasets. Neural Comp Appl. (2021) 33:14991–5025. 10.1007/s00521-021-06134-z

[9] Yang Z Xie L Zhou W Huo X Wei L Lu J. VoxSeP: semi-positive voxels assist self-supervised 3D medical segmentation. Multimedia Syst. (2023) 29:33–48. 10.1007/s00530-022-00977-9

[10] Shabani S Homayounfar M Vardhanabhuti V Mahani MAN Koohi-Moghadam M. Self-supervised region-aware segmentation of COVID-19 CT images using 3D GAN and contrastive learning. Comput Biol Med. (2022) 149:106033. 10.1016/j.compbiomed.2022.106033 36041270 PMC9419627

[11] Wang EK Chen CM Hassan MM Almogren A. A deep learning based medical image segmentation technique in Internet-of-Medical-Things domain. Fut Generat Comp Syst. (2020) 108:135–44. 10.1016/j.future.2020.02.05433460386

[12] Qayyum A Lalande A Meriaudeau F. Automatic segmentation of tumors and affected organs in the abdomen using a 3D hybrid model for computed tomography imaging. Comput Biol Med. (2020) 127:104097. 10.1016/j.compbiomed.2020.104097 33142142

[13] Saleem H Shahid AR Raza B. Visual interpretability in 3D brain tumor segmentation network. Comput Biol Med. (2021) 133:104410. 10.1016/j.compbiomed.2021.104410 33894501

[14] Wu Z Wei J Wang J Li R. Slice imputation: multiple intermediate slices interpolation for anisotropic 3D medical image segmentation. Comput Biol Med. (2022) 147:105667. 10.1016/j.compbiomed.2022.105667 35696751

[15] AlZu'bi S Shehab M Al-Ayyoub M Jararweh Y Gupta B. Parallel implementation for 3d medical volume fuzzy segmentation. Pattern Recognit Lett. (2020) 130:312–8. 10.1016/j.patrec.2018.07.026

[16] Bitarafan A Nikdan M Baghshah MS. 3D image segmentation with sparse annotation by self-training and internal registration. IEEE J Biomed Health Informat. (2020) 25:2665–72. 10.1109/JBHI.2020.3038847 33211667

[17] Liu C Si W Qian Y Liao X Wang Q Guo Y. Multipath densely connected convolutional neural network for brain tumor segmentation. In: Brainlesion: Glioma, Multiple Sclerosis, Stroke and Traumatic Brain Injuries: 4th International Workshop, BrainLes 2018, Held in Conjunction with MICCAI 2018, Granada, Spain, September 16, 2018, Revised Selected Papers, Part I 4. Springer (2019). p. 81–91.

[18] Rui-Qiang L Xiao-Dong C Ren-Zhe T Cai-Zi L Wei Y Dou-Dou Z. Automatic localization of target point for subthalamic nucleus-deep brain stimulation via hierarchical attention-UNet based MRI segmentation. Med Phys. (2023) 50:50–60. 10.1002/mp.15956 36053005

[19] Ou C Qian Y Chong W Hou X Zhang M Zhang X. A deep learning-based automatic system for intracranial aneurysms diagnosis on three-dimensional digital subtraction angiographic images. Med Phys. (2022) 49:7038–53. 10.1002/mp.15846 35792717

[20] Zhang J Xie Y Wang Y Xia Y. Inter-slice context residual learning for 3D medical image segmentation. IEEE Trans Med Imaging. (2020) 40:661–72. 10.1109/TMI.2020.3034995 33125324

[21] Wu Y Liao K Chen J Wang J Chen DZ Gao H. D-former: a u-shaped dilated transformer for 3d medical image segmentation. Neural Comput Appl. (2023) 35:1931–44. 10.1007/s00521-022-07859-1

[22] Hassanzadeh T Essam D Sarker R. Evolutionary deep attention convolutional neural networks for 2D and 3D medical image segmentation. J Digit Imaging. (2021) 34:1387–404. 10.1007/s10278-021-00526-2 34729668 PMC8669068

[23] Sun L Ma W Ding X Huang Y Liang D Paisley J. 3D spatially weighted network for segmentation of brain tissue from MRI. IEEE Trans Med Imaging. (2019) 39:898–909. 10.1109/TMI.2019.2937271 31449009

[24] Li C Chen W Tan Y. Point-sampling method based on 3D U-net architecture to reduce the influence of false positive and solve boundary blur problem in 3D CT image segmentation. Appl Sci. (2020) 10:6838. 10.3390/app10196838

[25] Liu X Yin R Yin J. Attention V-Net: a modified V-Net architecture for left atrial segmentation. Appl Sci. (2022) 12:3764. 10.3390/app12083764

[26] Indraswari R Kurita T Arifin AZ Suciati N Astuti ER. Multi-projection deep learning network for segmentation of 3D medical images. Pattern Recognit Lett. (2019) 125:791–7. 10.1016/j.patrec.2019.08.003

[27] Qayyum A Ahmad I Mumtaz W Alassafi MO Alghamdi R Mazher M. Automatic segmentation using a hybrid dense network integrated with an 3D-atrous spatial pyramid pooling module for computed tomography (CT) imaging. IEEE Access. (2020) 8:169794–803. 10.1109/ACCESS.2020.3024277

[28] Jiang Y Zhang Y Lin X Dong J Cheng T Liang J. SwinBTS: a method for 3D multimodal brain tumor segmentation using swin transformer. Brain Sci. (2022) 12:797. 10.3390/brainsci12060797 35741682 PMC9221215

[29] He R Xu S Liu Y Li Q Liu Y Zhao N. Three-dimensional liver image segmentation using generative adversarial networks based on feature restoration. Front Med. (2022) 8:794969. 10.3389/fmed.2021.794969 35071275 PMC8777029

[30] Sun D Wang Y Ni D Wang T. Autopath: image-specific inference for 3D segmentation. Front Neurorobot. (2020) 14:49. 10.3389/fnbot.2020.00049 32792934 PMC7393252

[31] Xu Y Gong M Chen J Chen Z Batmanghelich K. 3d-boxsup: Positive-unlabeled learning of brain tumor segmentation networks from 3d bounding boxes. Front Neurosci. (2020) 14:350. 10.3389/fnins.2020.00350 32410939 PMC7199456

[32] Gao W Li X Wang Y Cai Y. Medical image segmentation algorithm for three-dimensional multimodal using deep reinforcement learning and big data analytics. Front Public Health. (2022) 10:879639. 10.3389/fpubh.2022.879639 35462800 PMC9024167

[33] Li R Chen X. An efficient interactive multi-label segmentation tool for 2D and 3D medical images using fully connected conditional random field. Comput Methods Progr Biomed. (2022) 213:106534. 10.1016/j.cmpb.2021.106534 34839271

[34] Bennai MT Guessoum Z Mazouzi S Cormier S Mezghiche M. A stochastic multi-agent approach for medical-image segmentation: application to tumor segmentation in brain MR images. Artif Intell Med. (2020) 110:101980. 10.1016/j.artmed.2020.101980 33250150

[35] Hassanzadeh T Essam D Sarker R. 2D to 3D evolutionary deep convolutional neural networks for medical image segmentation. IEEE Trans Med Imaging. (2020) 40:712–21. 10.1109/TMI.2020.3035555 33141663

